# Anomaly Monitoring Method for Key Components of Satellite

**DOI:** 10.1155/2014/104052

**Published:** 2014-01-22

**Authors:** Jian Peng, Linjun Fan, Weidong Xiao, Jun Tang

**Affiliations:** ^1^College of Information Systems and Management, National University of Defense and Technology, Changsha 410073, China; ^2^Department of Telecommunications and Systems Engineering, University of Barcelona, 08202 Barcelona, Spain

## Abstract

This paper presented a fault diagnosis method for key components of satellite, called Anomaly Monitoring Method (AMM), which is made up of state estimation based on Multivariate State Estimation Techniques (MSET) and anomaly detection based on Sequential Probability Ratio Test (SPRT). On the basis of analysis failure of lithium-ion batteries (LIBs), we divided the failure of LIBs into internal failure, external failure, and thermal runaway and selected electrolyte resistance (*R*_*e*_) and the charge transfer resistance (*R*_ct_) as the key parameters of state estimation. Then, through the actual in-orbit telemetry data of the key parameters of LIBs, we obtained the actual residual value (*R*_*X*_) and healthy residual value (*R*_*L*_) of LIBs based on the state estimation of MSET, and then, through the residual values (*R*_*X*_ and *R*_*L*_) of LIBs, we detected the anomaly states based on the anomaly detection of SPRT. Lastly, we conducted an example of AMM for LIBs, and, according to the results of AMM, we validated the feasibility and effectiveness of AMM by comparing it with the results of threshold detective method (TDM).

## 1. Introduction

As primary means of human space exploration, aerospace technology is playing an increasingly important role in military, politics, economy, technology, and many other fields. Meanwhile, as highly requirement of satellite, the system of satellite becomes more and more complex. In such complex and huge investment system, there is an urgent need to improve the reliability and security of satellite system. Otherwise, a failure could lead to catastrophic consequences and enormous economic losses [1]. According to the statistics, the number of spacecrafts including satellite and space station had been launched is 764 between 1990 and 2001, but there are 121 spacecrafts that have been failed accounting for 15.8% of the total number of spacecrafts [2].

Fault diagnosis is an important problem that has been researched within diverse research areas and application domains. Many fault diagnosis techniques have been specifically developed for certain application domains, while others are more generic [3]. According to the view of German fault diagnosis authority Professor P. M. Frank, fault diagnosis methods can be divided into three kinds: (1) based on knowledge method; (2) based on signal process method; (3) based on analytical model method.

As rapidly developing artificial intelligence and computer technology, it provides a new theoretical foundation to fault diagnosis. As a result, the knowledge-based fault diagnosis method (KFDM) shows strong vitality and superiority. Moreover, because of its intelligence, the KFDM is becoming the focus of fault diagnosis and has made a lot of achievements. In accordance with the intelligent method used, the KFDM can be divided into based-on-neural-network fault diagnosis method (NNFDM), based-on-expert-system fault diagnosis method (ESFDM), based-on-consistency-checking fault diagnosis method (CCFDM), and based-on-qualitative-model fault diagnosis method (QMFDM). Although the NNFDM [4–7] requires a lot of historical data to train network prior, it needs to address the problems of data collection. In addition, the scale of neural network (number of layers and hidden nodes) is always determined by experiment or experience, which does not have mature theoretical guidance. The serious drawback of ESFDM [8, 9] is that it only can detect predictable failure, and the bottleneck of its diagnosis ability is the source of expert knowledge. Moreover, there is a logical reasoning “combinatorial explosion” problem. The drawbacks of CCFDM [10–12] are (1) when dealing with uncertain situations, its adaptive ability is very poor and bring poor false alarm rate and missed alarm rate; (2) the preset logic decision is not necessarily the optimal approach; (3) the production rules can only express predictable failure. The drawbacks of QMFDM [13–19] are: (1) the accuracy of diagnosis entirely depends on the model; (2) the potential failure diagnosis capability is weak; (3) the models currently used are discrete model, so first of all the successive system model should be discretized, which would affect the accuracy of diagnosis. In a word, the KFDM is not suitable for key components of satellite.

According to the input and output signals of system which directly can be measured, the based on signal processing fault diagnosis method (SPFDM) [21–25] finds out the relationship between these signals and the source of failures. The SPFDM extracts the amplitude, phase, spectral, and any other characteristic values by using the related functions, higher-order statistics, spectrum, autoregressive moving average, wavelet technology, and so on and detects the anomalies with these characteristic values. The advantages of SPFDM are that it is easy to be achieved and has good effectiveness, but the disadvantage of SPFDM is that the ability to detect the potential early anomaly is not enough. So the SPFDM is also not suitable for key components of satellite.

The based on analytical model fault diagnosis method (AMFDM) [26–29] is the earliest and most thorough, most proven methods, which can be divided into state estimation method, equivalent space method, and parameter estimation method. However, due to the fact that the particularity and complexity of space environment are complex and other uncertain factors are numerous, it is difficult to establish an accurate analytical model; as a result the equivalent space method and parameter estimation method are unsuitable for the fault diagnosis problems of satellite. Therefore, there is only the state estimation method that can solve the fault diagnosis problems of satellite. Multivariate State Estimation Techniques (MSET) [30] is a nonlinear, nonparametric modeling method that was originally developed by Argonne National Laboratory (ANL) for high-sensitivity proactive fault monitoring applications in commercial nuclear power applications. Some papers [31, 32] had used the MSET to detect the anomaly of internal combustion engine, electronic products, and so on. Nevertheless, the MSET is not applied in the fault diagnosis of satellite so far. In view of the fact that the data of key components of satellite is too few, it is difficult to obtain large amount of historical data and sufficient knowledge on abnormal components and rules. Consequently, the traditional based-on-analysis model methods are not suitable for fault diagnosis of satellite.

Satellite power system is the heart of entire satellite system, whose mission is to provide electrical power to the satellite, as well as to store, distribute, and control the electrical power [33]. Because of its superior weight ratio of the energy, the volume ratio of the energy, and cycle life performance, lithium-ion batteries (LIBs) have big advantages in comparison to the traditional energy storage batteries, such as nickel-cadmium battery and nickel-hydrogen battery.

To sum up, grounded on the fault diagnosis problems of LIBs in satellite, we propose a fault diagnosis method for key components of satellite, which is called Anomaly Monitoring Method (AMM), based on study of Multivariate State Estimation Techniques (MSET) and Sequential Probability Ratio Test (SPRT). The AMM is made up of state estimation based on MSET and anomaly detection based on SPRT, which is developed by improving MSET and creatively combining MSET and SPRT.

The main contributions of this paper are as follows.

- We propose a fault diagnosis method for key components of satellite, which is called Anomaly Monitoring Method (AMM), and it is made up of state estimation based on MSET and anomaly detection based on SPRT.
- On the basis of the study of MSET, we improve the method of MSET, considering the fact that it is difficult to obtain large amount of historical data and sufficient knowledge on abnormal components and rules.
- According to practical engineering applications, we detailed introduce the key technologies of state estimation based on MSET (i.e., data normalization process, selection of training data *T*, selection and optimization of nonlinear operator, and structure of memory matrix *D*).
- We conduct quantitative measurement experiment to validate the feasibility and effectiveness of AMM by comparing it with the result of threshold detective method (TDM).

The rest of this paper is organized as follows. The analysis failure of LIBs is presented in Section 2.  Section 3 discusses the state estimation based on MSET.  Section 4 describes the anomaly detection based on SPRT.  Section 5 provides the result of AMM and compares it with the result of TDM.  Section 6 presents the conclusion and recommendations for future work.

## 2. Analysis Failure of LIBs

Compared with the traditional batteries, such as nickel-cadmium battery and nickel-hydrogen battery, LIBs are different from the general chemistry power supply, whose charging and discharging operations are done by insertion and extraction of their positive and negative. When the LIBs are charging, the positive electrode releases the lithium-ion in the electrolyte and this process is called extraction. And then the negative electrode intakes the lithium-ion from the electrolyte and this process is called insertion. Similarly, when the LIBs are discharging, the procedure occurs contrary to the above process. Therefore, due to the iterative insertion and extraction of lithium-ion, the process of charge and discharge of LIBs seems like a swaying rocking chair, that is why the LIBs are always called “rocking chair battery.”

On the basis of analysis failure of LIBs, we know that the failure of LIBs can be divided into internal failure, external failure, and thermal runaway. The internal failure is caused by three main aspects [34]: (1) degradation of the electrode performance; (2) loss of the electrolyte; (3) aging of the diaphragm. These previous aspects have resulted in the increase of internal resistance which directly leads to the LIBs failure. But the external failure of LIBs is the result of many factors. Not only the effect of the structure, but also the method of usage. And the involving factors are the use procedure, the environment (temperature-sensitive), the charge and discharge mechanism, the lithium-ion electric stress, and so on. As the thermal reaction between the electrolyte and the electrode material, which is called “thermal runaway,” always leads to a devastating accident, the safety floor of LIBs is determined by their initial temperature [34]. According to the analysis of the internal failure, external failure, and thermal runaway of LIBs, the key performance parameters of LIBs are internal resistance, discharge current, operating voltage, and temperature. However, LIBs as an energy carrier can be equivalent to a constant voltage source and a resistor in the ideal electrical calculations. The study shows that the status and performance of LIBs are closely related to the impedance change [34].

**Figure 1:**
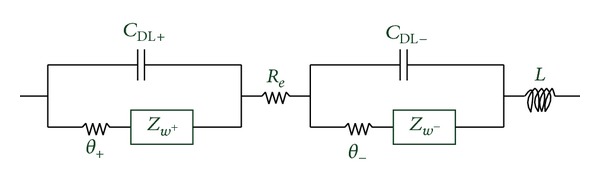
LIBs impedance equivalent circuit model.

Therefore, the study of impedance of LIBs has become a hot issue in manufacturing, testing, and surveillance of LIBs. Through the working principle of LIBs research, the LIBs' impedance equivalent circuit model is shown in Figure 1, where *L* represents the inductance, *R*_*e*_ represents the electrolyte resistance, *θ* represents the charge transfer resistance, *C*_DL_ represents the capacitance of the electrode polarization which is the electric double layer capacitor, and *Z*_*w*_ represents the Warburg impedance. In general, *θ*_+_ and *θ*_−_ can be represented by *R*_ct_. So the internal resistance of the LIBs can be represented by the electrolyte resistance (*R*_*e*_) and the charge transfer resistance (*R*_ct_). In practical engineering applications, *R*_*e*_ and *R*_ct_ can be measured by electrochemical impedance spectroscopy method (EIS).

Therefore, according to analysis failure of LIBs, we select *R*_*e*_ and *R*_ct_ as the key parameters of state estimation based on MSET.

## 3. State Estimation Based on MSET

**Figure 2:**
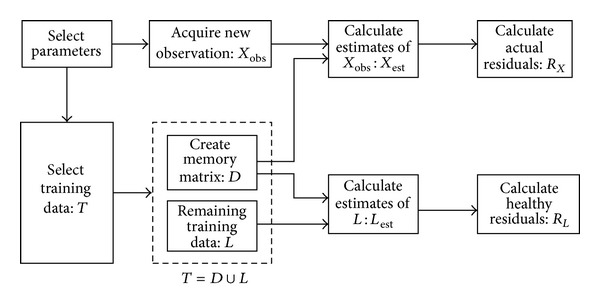
State estimation process based on MSET [20].

MSET is an advanced pattern recognition technology, which achieves the state estimation by measuring the similarity among various monitoring parameters in the normal operating range. The basic process of state estimation based on MSET is shown in Figure 2.

### 3.1. Steps of State Estimation

The basic steps of state estimation based on MSET can be roughly described as follows.

**Step 1 (get observation matrix of monitoring parameters)**

**Figure 3:**
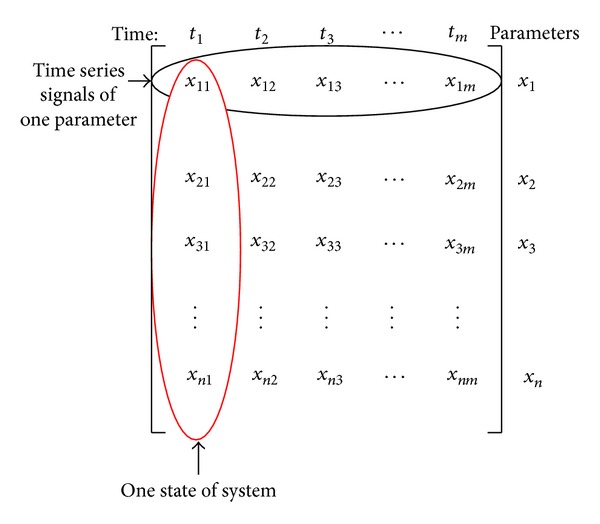
Actual in-orbit telemetry data matrix in MSET [20].

On the basis of actual in-orbit telemetry data, the observation matrix of monitoring parameters is selected from it, which is denoted by *X*_obs_. The observation matrix *X*_obs_ is a matrix of *n* × *m*, where *n* represents the number of monitoring parameters and *m* represents the number of the states. The actual in-orbit telemetry data matrix defined by MSET is shown in Figure 3.

**Step 2 (select training data)**

The Training data is a matrix that consists of many healthy historic states, which is denoted by the letter *T*. The training data *T* is a matrix of *n* × *m*_1_, where *n* also represents the number of monitoring parameters and *m*_1_ represents the number of the healthy states. The selection requirements of training data *T* are (1) the states of training data *T* must include the dynamic change states of monitoring parameters; (2) the training data *T* cannot contain any unhealthy states.

**Step 3 (create memory matrix)**

The memory matrix is selected from training data *T* in accordance with appropriate selecting rules, which is denoted by the letter *D*. The memory matrix *D* is a matrix of *n* × *k*_1_, where *n* represents the number of monitoring parameters and *k*_1_ represents the number of the states contained in the memory matrix *D*.

The steps of create memory matrix *D* are as follows:

1. artificially determine *k*_1_ according to the number of monitoring parameters;
2. select the extreme states of all monitoring parameters from training data *T*. If the number of extreme states is equal to *k*_1_, then the memory matrix *D* is completely created; otherwise, go to (3);
3. if the number of extremely states is less than *k*_1_ (the number of extremely states cannot be more than *k*_1_), then continue to follow the next selection rules: firstly, calculate the Euclidean norm of the remaining training data *T* and then get the vector *T*_1_; secondly, arrange the vector *T*_1_ by descending or ascending, and then get the vector *T*_2_; lastly, according to equidistant sampling method, select the remaining states of memory matrix *D* from the vector *T*_2_.

**Step 4 (create remaining training data)**

After the memory matrix *D* has been selected, those nonselected states in the training data *T* form a new matrix called remaining training data which is defined as the letter *L*. The remaining training data *L* is a matrix of *n* × *k*_2_, where *n* represents the number of monitoring parameters and *k*_2_ is the number of the states contained in the remaining training data *L*. So the remaining training data *T*, the memory matrix *D*, and the remaining training data *L* have the following equation relationship:

(1) $$\begin{array}{c} \begin{array}{ccccc} T & = & D & \cup & L\\ n\times k & & n\times k_{1} & & n\times k_{2}. \end{array} \end{array}$$

**Step 5 (calculate estimation matrix for the observation matrix *X*_*obs*_)**

The estimate of observation matrix *X*_obs_, defined as *X*_est_, is the expected value calculated from the healthy data. This estimate has the same data format as the observation, which is an *n*-element vector that is thought to be the weighted (linear) combination of states in memory matrix *D*. The calculation equation is

(2) $$\begin{array}{c} X_{\text{est}}=D∙W, \end{array}$$

where *W* is a weight vector, which decides a similarity measure between each state in memory matrix *D* and the estimation matrix *X*_obs_. The formula for this vector is derived from the least square method by minimizing the error vector:

(3) $$\begin{array}{c} \varepsilon =X_{\text{obs}}-X_{\text{est}}. \end{array}$$

when the *ε* is constrained by minimization (set ∂*ε* ^2^/∂*W* = 0), the least square error estimation solution of weight vector *W* is:

(4) $$\begin{array}{c} W={\left(D^{T}∙D\right)}^{-1}∙\left(D^{T}∙X_{\text{obs}}\right). \end{array}$$

So through (2), (3), and (4), the estimation matrix *X*_est_ for the observation matrix *X*_obs_ is

(5) $$\begin{array}{c} X_{\text{est}}=D∙{\left(D^{T}∙D\right)}^{-1}∙\left(D^{T}∙X_{\text{obs}}\right). \end{array}$$

**Step 6 (calculate the estimation matrix of the remaining training data *L*)**

Similarly, the estimation matrix for the remaining training data *L* defined as *L*_est_, which is the estimates of all the remaining data *L*. The calculation equation is

(6) $$\begin{array}{c} L_{\text{est}}=D∙W^{\prime }, \end{array}$$

where *W*′ is also a weight vector, which decides a similarity measure between each state in memory matrix *D* and the estimation matrix *L*_est_. The same as above, the estimation matrix *L*_est_ for the remaining training data *L* is

(7) $$\begin{array}{c} L_{\text{est}}=D∙{\left(D^{T}∙D\right)}^{-1}∙\left(D^{T}∙L\right). \end{array}$$

**Step 7 (calculate residual value)**

MSET then calculates the residual value between the estimates (*L*_est_) and remaining training data *L*. Because all these remaining training data *T* are healthy, the residual value presents the features of healthy states of the product and is called healthy residual, which is defined as *R*_*L*_. MSET also calculates the residual value between the estimates (*X*_est_) and the observation matrix *X*_obs_; this residual value shows the actual states of the product and is called actual residual, which is defined as *R*_*X*_. The definition of residual value is the value of the estimated value minus the actual value. Therefore, the calculation equations of the actual residual *R*_*X*_ and healthy residual *R*_*L*_ are

(8) $$\begin{array}{c} R_{X}=X_{\text{est}}-X_{\text{obs}}{,\;\;R}_{L}=L_{\text{est}}-L. \end{array}$$

In summary, through the introduction above, the basic steps of the state estimates based on MSET are clear. But in practical engineering applications, the state estimation based on MSET is more complex than the above-mentioned steps, which involves, in the data normalization process, the selection of training data *T*, the selection and optimization of nonlinear operator, and the structure of memory matrix *D*. So next there is a detailed introduction of the state estimation based on MSET in practical engineering applications.

### 3.2. Data Normalization Process

In practical engineering application, the selected parameters of product might not be the same order of magnitude. And due to the fact that the weight vector *W* represents a similarity measure between the observation matrix *X*_obs_ and the memory matrix *D*, the value of weight vector *W* is completely determined by high order of magnitude parameter data, such as selecting *R*_*e*_, *R*_ct_, and capacity as the key parameters, shown in Table 1. Obviously, the internal resistance data and the capacity data are clearly not the same order of magnitude. So at this time the value of weight vector *W* is completely determined by the capacity data, and the internal resistance data have lost the meaning of existence. Moreover, according to Section 3.4, if the observation matrix *X*_obs_ is not normalized, the nonlinear operator is not available to be optimized. So, in order to solve these problems, the data of each monitoring parameter firstly should be normalized, just as shown in Table 2.

### 3.3. Selection and Update of Training Data *T*

**Figure 4:**
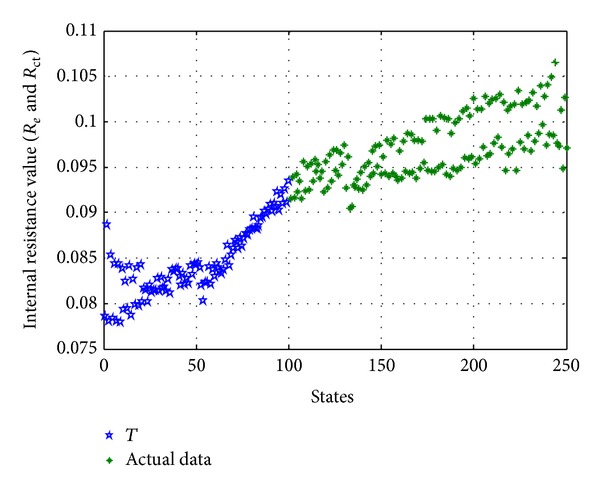
Selection of training data *T* from the actual in-orbit telemetry data.

According to the introduction of training data *T* in Section 3.1, the training data *T* is a matrix that consists of many healthy historic states. However, in practical engineering applications, LIBs of satellite do not have historical healthy data. Therefore, the traditional MSET be cannot suitable for the fault diagnosis of satellite in practical engineering applications. In order to resolve this problem, we improve the MSET on the basis of the traditional MSET. So we select the training data *T* from the actual in-orbit telemetry data and consider the initial data of the actual in-orbit telemetry data as the training data *T*, shown in Figure 4. But in order to ensure that the states contained in training data *T* are healthy states, the selected portion of states should be small. And then due to the fact that the trading data *T* selected originally does not contain all dynamic change states of monitoring parameters, the trading data *T* selected originally should be updated. There are two methods for training data *T* update.

**Figure 5:**
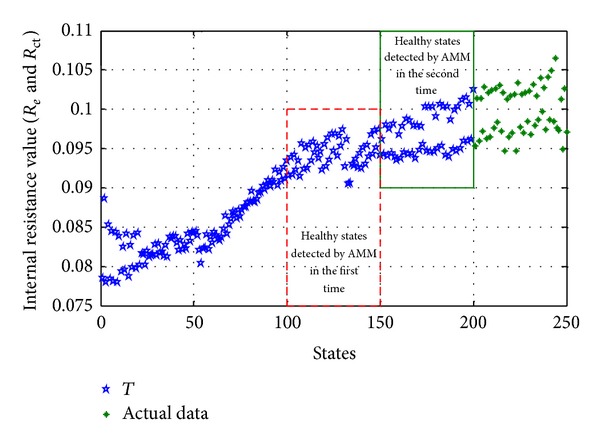
Update of training data *T* with nonquantitative update method.

**Figure 6:**
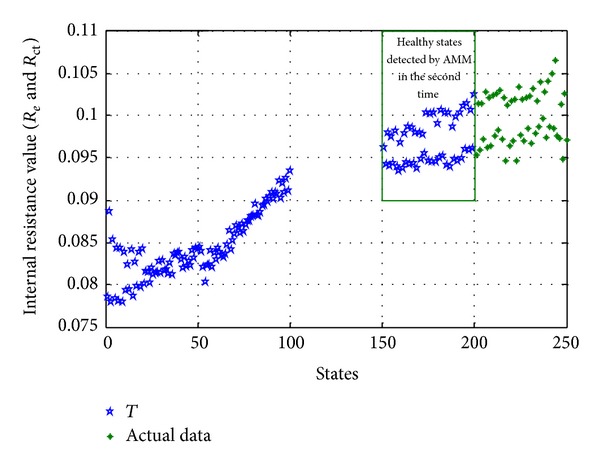
Update of training data *T* with quantitative update method.

1. *Nonquantitative Update Method*. With the continuation of anomaly detection, get the healthy states detected by AMM and add them to the training data *T* selected originally. As shown in Figure 5, the (101–150) states are the healthy states detected by AMM in the first time and the (151–200) states are the healthy states detected by AMM in the second time, and get all the healthy states (101–200) detected by AMM and add them to the training data *T*.
2. *Quantitative Update Method*. With the continuation of anomaly detection, only get the healthy states in the floating window every time detected by AMM and add them to the training data *T* selected originally. As shown in Figure 6, the (151–200) states are the healthy states detected by AMM in the second time, and only get the healthy states (151–200) detected by AMM in the second time and add them to the training data *T* selected originally.

Comparing the two update methods, the more states contained in the nonquantitative update method, the better state estimation effect of the nonquantitative update method might be. However, in practical engineering applications, the actual in-orbit telemetry data of LIBs is extremely large, so the calculated amount of the nonquantitative update method would be vast. Actually by using the quantitative update method, not only the training data *T* reaches the purpose that contains dynamic change states of monitoring parameters but also the amount of calculation will be greatly reduced. Therefore, considering the calculated amount in practical engineering applications, the quantitative update method is better than the nonquantitative update method.

### 3.4. Selection and Optimization of Nonlinear Operator

Through (4), we know that if the weight vector *W* is existent, the *D* ^*T*^∙*D* must be reversible. However, a necessary but not sufficient condition of the reversible *D* ^*T*^∙*D* is that the number of columns of the memory matrix *D* should be less than the number of lines of the memory matrix *D*; in other words, the number of states contained in memory matrix *D* should be less than the number of monitoring parameters. However, in practical engineering applications, this condition is very difficult to be fulfilled. In order to provide adequate statistical information of monitoring parameters, the memory matrix *D* must include a large number of states. So the number of states contained in memory matrix *D* cannot be less than the number of monitoring parameters. Actually the *D* ^*T*^∙*D* cannot be reversible, that is because of the correlation of different states contained in the memory matrix *D*. Therefore, in order to solve this problem in practical engineering applications, MSET method introduction of the nonlinear operator is defined as ⊗, which is a typical operator that calculates normalized similarity between different data vectors.

The most common nonlinear operators are [35]

1. the Euclidean norm ($f(x,y)=\sqrt{\sum_{m=1}^{k}{\left(x_{m}-y_{m}\right)}^{2}}$),
2. city block distance (*f*(*x*, *y*) = ∑_*m*=1_^*M*^|*x*_*m*_ − *y*_*m*_|),
3. linear correlation coefficient ($f(x,y)=\sum_{m=1}^{M}\left(x_{m}-\overset{-}{x})(y_{m}-\overset{-}{y}\right)/\sqrt{\sum_{m=1}^{M}{\left(x_{m}-\overset{-}{x}\right)}^{2}\sum_{m=1}^{M}{\left(y_{m}-\overset{-}{y}\right)}^{2}}$),
4. the Gaussian operator, and so on.

In this paper, we use the most commonly used nonlinear operator—the Gaussian operator. The Gaussian operator equations can be described as follows:

(9) $$\begin{array}{c} f\left(x,y,h\right)=\overset{M}{\underset{m=1}{\sum }}\frac{1}{\sqrt{2\pi }h}e^{{\left(x_{m}-y_{m}\right)}^{2}/2h^{2}}, \end{array}$$

where *h* is the filter coefficient or also known as bandwidth.

Then, through the selection of nonlinear operator, (4), (5), and (7) can be described as follows:

(10) $$\begin{array}{c} W={\left(D^{T}⊗D\right)}^{-1}∙\left(D^{T}⊗X_{\text{obs}}\right),\\ X_{\text{est}}=D∙{\left(D^{T}⊗D\right)}^{-1}∙\left(D^{T}⊗X_{\text{obs}}\right),\\ L_{\text{est}}=D∙{\left(D^{T}⊗D\right)}^{-1}∙\left(D^{T}⊗L\right). \end{array}$$

**Figure 7:**
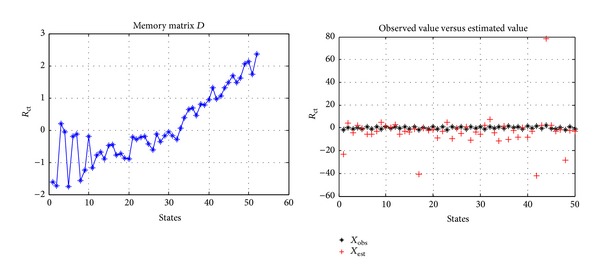
Memory matrix *D* with strong correlation.

However, if the states contained in the memory matrix *D* have strong correlation, the state estimation will be very badly. This is because the cardinal number of the (*D* ^*T*^⊗*D*)^−1^ will become tremendous, and then the estimated value will also become tremendous. So, under these circumstances, the state estimation based on MSET is equivalent of a magnifying glass. If the observation matrix *X*_obs_ includes noise, then it is difficult to obtain a stable state estimation, and this restricted memory matrix *D* is like a noise amplifier, just as shown in Figure 7.

**Figure 8:**
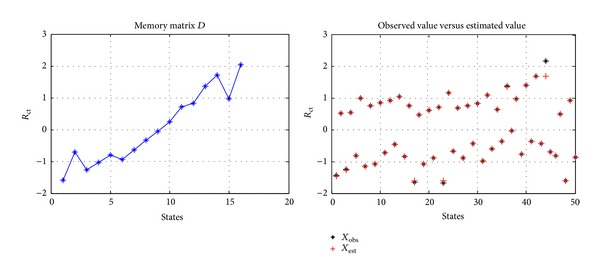
Memory matrix *D* without strong correlation.

If the states contained in memory matrix *D* do not have strong correlation, the state estimation based on MSET will be good, and the estimated value will not amplify the noise, just as shown in Figure 8.

In summary, if the observation matrix *X*_obs_ includes noise, the states contained in memory matrix *D* could not have strong correlation when using the nonlinear operator; otherwise, the state estimation based on MSET will be like a noise amplifier. However, in practical engineering applications, the observation matrix *X*_obs_ inescapable includes some noise and the states contained in memory matrix *D* cannot be completely without strong correlation. So, in order to resolve this problem, we introduced the ridge regularization when inverse the (*D* ^*T*^ ⊗ *D*). And then after that, (10) can be described as follows:

(11) $$\begin{array}{c} W={\left(D^{T}⊗D+\lambda I\right)}^{-1}∙\left(D^{T}⊗X_{\text{obs}}\right),\\ X_{\text{est}}=D∙{\left(D^{T}⊗D+\lambda I\right)}^{-1}∙\left(D^{T}⊗X_{\text{obs}}\right),\\ L_{\text{est}}=D∙{\left(D^{T}⊗D+\lambda I\right)}^{-1}∙\left(D^{T}⊗L\right), \end{array}$$

where *λ* is the ridge regularization parameter (*λ* > 0) and *I* is the identity matrix.

The optimization problem of *λ* is a one-dimensional optimization problem, which can be solved by using nonlinear methods, such as conjugate gradient descent and so on. When the data of each monitoring parameter follow normal distributions with mean 0 and variance 1, the optimization bandwidth is *h* = 1 and the optimization ridge regularization is *λ* = 1 [36].

### 3.5. Structure of Memory Matrix *D*

On the basis of the introduction of creating memory matrix *D* in Section 3.1, the selection of memory matrix *D* can be roughly divided into two steps:

1. determine the number of states contained in the memory matrix *D*, *k*_1_;
2. select *k*_1_ states from the training data *T* with appropriate rules.

The most important steps of creating memory matrix *D* is to determine the number of states contained in the memory matrix *Dk*_1_ which is closely related to the estimation performance. Generally, estimates produced by using a lesser number of states contained in the memory matrix *D* tend to be less accurate and less reliable than those created by using more states. Under some circumstances, using too many states can capture signal noise and lead to undesirable effects (the “overtraining” syndrome), just as shown in Figure 7. In addition, consideration of the spend time of state estimation, the spend time of state estimation based on MSET is closely related to the number of states contained in the memory matrix *D*, that is, the more states contained in the memory matrix *D*, the longer time spent by state estimation [31].

On the other hand, the selection of memory matrix *D* is directly related to the selection of remaining training data *L*, according to (1). So if the number of states contained in the memory matrix *Dk*_1_ is too big, it will inevitably lead to the fact that the number of states contained in the remaining training data *Lk*_2_ is too small, which is obviously not conducive to the SPRT detection.

To sum up, there is a basic principle for the determination of the number of states contained in the memory matrix *D*: the states contained in the memory matrix *D* should be as few as possible when the states cover all the dynamic range of monitoring parameters.

## 4. Anomaly Detection Based on SPRT

Through the state estimation based on MSET, the actual residual value and healthy residual value have been obtained, and then the anomaly detection can be going based on these residual values. SPRT is a common technique for binary hypothesis testing, which has been widely used to conduct binary hypothesis testing in many applications. Wald's SPRT [37] was developed based upon two assumptions: (1) samples are independent and identically distributed; (2) samples follow a priori known distribution function. As the SPRT method can detect not only the anomaly states outside the threshold but also the anomaly state in the threshold, so in this paper we detect the anomaly states based on SPRT.

However, the actual residual value and healthy residual value obtained by MSET are both matrices. So in view of this situation this paper puts forward a preprocessing method for the residual values before anomaly detection based on SPRT: reduce the dimension of the actual residual value and healthy residual value by introducing a weight vector *X* = [*x*_1_, *x*_2_,…*x*_*n*_], where *x*_*i*_ is the weight ratio of the monitoring parameter *i* on product performance, just as follows:

(12) RX′=X∙RX=[x1,x2,…,xn]∙[y11y12⋯y1my21y22⋯y2m⋮⋮⋯⋮yn1    yn2⋯ynm]=[y1,y2,…,ym],RL′=X∙RL=[x1,x2,…,xn]∙[y11′y12′⋯y1m′′y21′y22′⋯y2m′′⋮⋮⋯⋮yn1′yn2′⋯ynm′′]=[y1′,y2′,…,ym′′],

where *y*_*ij*_ and *y*_*ij*_′ represent the residual value of the actual residual value and the healthy residual value separately when the monitoring parameter *i* is on the state *j* and *y*_*i*_ and *y*_*i*_′ represent the residual value of the actual residual value (*R*_*X*_′) and the healthy residual value (*R*_*L*_′) after reducing the dimension separately on the state *i*.

The actual residual value can be tested by mean and variance based on SPRT when the residuals (*R*_*X*_′ and *R*_*L*_′) obey normal distribution. Then set the original hypothesis to be *H*_0_, and the alternative hypothesis is any one of *H*_1_ ~ *H*_4_:

(13) H0: mean    μ0,variance  σ02,H1: mean    μ1>μ0,variance  σ02,H2: mean    μ2<μ0,variance  σ02,H3: mean    μ0,variance  σ02∙V,H4: mean    μ0,variance  σ02V,

where *V* is the variance factor, *μ*_0_ and *σ*_0_ are the mean and variance of healthy residual value, *μ*_1_ and *μ*_2_ are the mean of anomaly states, *H*_1_ and *H*_2_ are the alternative hypothesis for the mean test, and *H*_3_ and *H*_4_ are the alternative hypothesis for the variance test.

In the classic Wald SPRT method, a sequential probability ratio is constructed as

(14) $$\begin{array}{c} \Lambda \left(Y\right)=\frac{F_{i}\left(Y_{n}\;|\;H_{i}\right)}{G\left(Y_{n}\;|\;H_{0}\right)}, \end{array}$$

where *F*_*i*_(*Y*_*n*_ | *H*_*i*_) is the likelihood function for observing the sample sequence *Y*_*n*_ if the hypothesis *H*_*i*_ is true and *G*(*Y*_*n*_ | *H*_0_) is the likelihood function for observing the sample sequence *Y*_*n*_ if the hypothesis *H*_0_ is true. Deciding which hypothesis to accept is determined by comparing the ratio Λ(*Y*) with some upper and lower bound values, which being related to *α* and *β*, by the following rules:

1. if Λ(*Y*_*n*_) ≥ *B*≅(1 − *β*)/*α*, accept *H*_*i*_;
2. if Λ(*Y*_*n*_) ≤ *A*≅*β*/(1 − *α*), accept *H*_0_;
3. if *A* < Λ(*Y*_*n*_) < *B* cannot make a decision, increase the sample size to *n* + 1 and then repeat the comparison,

where *α* is called prescribed first kind of error and *β* is called prescribed second kind of error, and in practical engineering applications, *α* and *β* are also called the prescribed false alarm rate and prescribed miss alarm rate, respectively.

Next, the mean test of actual residual based on SPRT can be detailed described and as follows.

According to (13), we can get that the original hypothesis is *H*_0_, and the alternative hypothesis is *H*_1_. And then the likelihood function of actual residual can be described as follows:

(15) G(Y)=g1(y)g2(y)⋯gn(y)=1(2πσ02)n/2exp⁡[−12σ02∑i=1n(yi−μ0)2],F1(Y)=f1(y)f2(y)⋯fn(y)=1(2πσ02)n/2exp⁡[−12σ02∑i=1n(yi−μ1)2].

So according to (14), the likelihood ratio Λ(*Y*) is

(16) Λ(Y)=Fi(Yn ∣ Hi)G(Yn ∣ H0)=exp⁡[(μ1−μ0)σ02∑i=1n(yi)+n2σ02(μ12−μ02)].

And then the logarithmic function of the likelihood ratio Λ(*Y*) is

(17) $$\begin{array}{c} \ln\Lambda \left(Y\right)=\left[\frac{\left(\mu_{1}-\mu_{0}\right)}{\sigma_{0}^{2}}\overset{n}{\underset{i=1}{\sum }}\left(y_{i}\right)+\frac{n}{2\sigma_{0}^{2}}\left({\mu_{1}}^{2}-\mu_{0}^{2}\right)\right]. \end{array}$$

Lastly, let ln⁡Λ(*Y*) substitute in the decision expression of SPRT (ln⁡*A* < ln⁡Λ(*Y*) < ln⁡*B*), namely,

(18) ln⁡β1−α<[(μ1−μ0)σ02∑i=1n(yi)+n2σ02(μ12−μ02)]<ln⁡1−βα.

The meaning of the *n* in the upper equations does not represent the total length of the test sample but represents the length of the test sample when the decision is done.

When ln⁡Λ(*Y*) has been calculated, the SPRT Index of residual sample can be calculated, and the calculation steps are as follows.

**Step 8**

Set the logarithmic of the likelihood ratio of each state to be equal to *s*(*i*).

**Step 9**

If *s*(*i*)⩾ln⁡*B*, then set *s*_1_(*i*) = ln⁡*B*; if *s*(*i*) ⩽ ln⁡*A*, then set *s*_1_(*i*) = ln⁡*A*; if ln⁡*A* < *s*(*i*) < ln⁡*B*, then set *s*_1_(*i*) = *s*(*i*), where *A* is the lower limit and *B* is the upper limit.

**Step 10**

If *s*_1_(*i*) > 0, then set *s*_2_(*i*) = *s*_1_(*i*)/ln⁡*B*, or set *s*_2_(*i*) = *s*_1_(*i*)/ln⁡*A*.

**Step 11**

**Figure 9:**
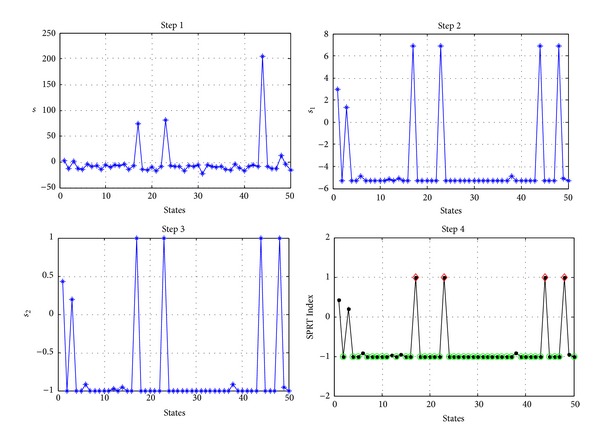
Calculation steps of SPRT Index.

Then the *s*_2_(*i*)∈[1  1], where *s*_2_(*i*) = 1 corresponds to the abnormal state and indicated by the diamond in the figure, where *s*_2_(*i*) = −1 corresponds to the healthy state and indicated by the circle in the figure, where −1 < *s*_2_(*i*) < 1 corresponds to the state that is unable to be determined and indicated by the dot in the figure. The steps of calculation also can be orderly expressed in Figure 9.

## 5. AMM Applications for LIBs

The AMM consists of state estimation based on MSET and anomaly detection based on SPRT. In Section 3, we have detailed introduction of the steps of LIBs' state estimation based on MSET. In Section 4, according to the actual residual value and healthy residual value of LIBs that are got by state estimation, we have detailed illustration of the mean test of actual residual based on SPRT. Next, we conduct an example of AMM for LIBs, and, according to the result of AMM, we validate the feasibility and effectiveness of AMM by comparing it with the result of TDM.

### 5.1. State Estimation for LIBs

**Figure 10:**
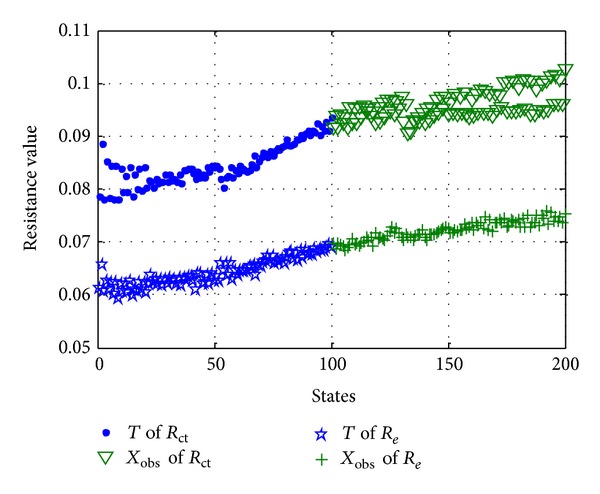
Training data *T* and observation matrix *X*_obs_ of *R*_*e*_ and *R*_ct_.

According to the steps of state estimation based on MSET, first of all is to select parameters and get observation matrix of monitoring parameters *X*_obs_. In Section 2, according to analysis failure of LIBs, we select *R*_*e*_ and *R*_ct_ of LIBs as the key parameters of state estimation based on MSET. And then, on the basis of actual in-orbit telemetry data of LIBs, we get observation matrix *X*_obs_ of *R*_*e*_ and *R*_ct_. Meantime, select training data *T* from actual in-orbit telemetry data of LIBs according to the introduction of Section 3.3, and the training data *T* and observation matrix *X*_obs_ of *R*_*e*_ and *R*_ct_ are shown in Figure 10, where dot represents the training data *T* of *R*_ct_ triangle represents the observation matrix of *R*_ct_, five-pointed star represents the training data *T* of *R*_*e*_, and plus sign represents the observation matrix of *R*_*e*_.

**Figure 11:**
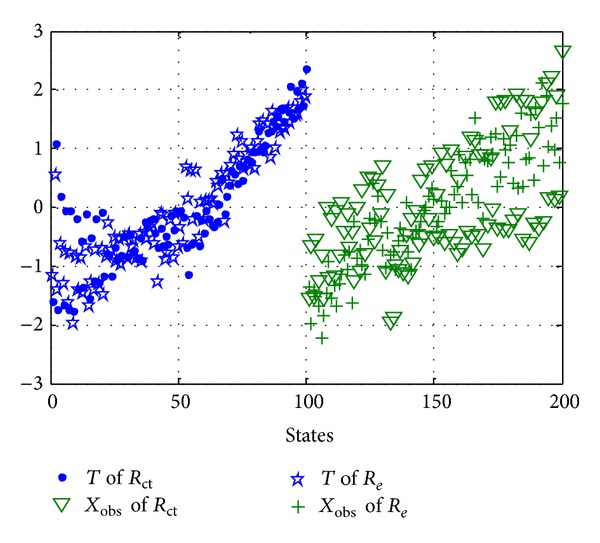
Training data *T* and observation matrix *X*_obs_ of *R*_*e*_ and *R*_ct_ after normalization process.

Secondly, according to the introduction of data normalization process in Section 3.2, the training data *T* and observation matrix *X*_obs_ of *R*_*e*_ and *R*_ct_ should be normalized with mean 0 and variance 1 of normal distribution before state estimation, so the data after normalization process are shown in Figure 11, where *Y* label represents the value of resistance value after normalization process.

**Figure 12:**
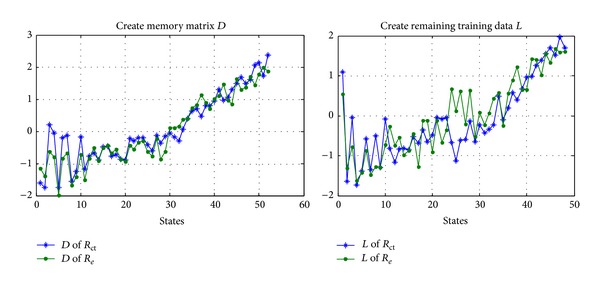
Construction of memory matrix *D* and the remaining training data *L* of *R*_*e*_ and *R*_ct_.

Thirdly, along with the steps of state estimation based on MSET, we create memory matrix *D* and remaining training data *L* of *R*_*e*_ and *R*_ct_ from training data *T* in Figure 11, just as shown in Figure 12, where the left figure is the memory matrix *D* of *R*_*e*_ and *R*_ct_ and the right figure is the remaining training data *L* of *R*_*e*_ and *R*_ct_.

**Figure 13:**
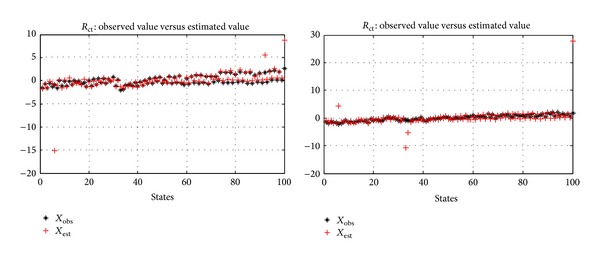
Comparison of the observed value and estimated value of *R*_*e*_ and *R*_ct_.

And then through the construction of memory matrix *D* and the remaining training data *L* of *R*_*e*_ and *R*_ct_ in the upper figure and the observation matrix *X*_obs_ in Figure 11, the weight vector *W*, the estimation matrix of observation matrix *X*_obs_, *X*_est_, and the estimation matrix of remaining training data *L*, *L*_est_ can be calculated. Just as shown in Figure 13, the left figure is the comparison of observed value and estimated value of *R*_ct_ and the right figure is the comparison of observed value and estimated value of *R*_*e*_.

**Figure 14:**
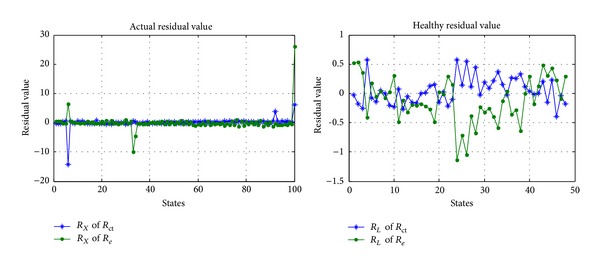
Actual residual value and healthy residual value of *R*_*e*_ and *R*_ct_.

Lastly, the actual residual value *R*_*X*_ and healthy residual value *R*_*L*_ of *R*_*e*_ and *R*_ct_ can be calculated by MSET, just as shown in Figure 14, where left figure is the actual residual value of *R*_*e*_ and *R*_ct_ and right figure is the healthy residual value of *R*_*e*_ and *R*_ct_.

### 5.2. Anomaly Detection for LIBs

**Figure 15:**
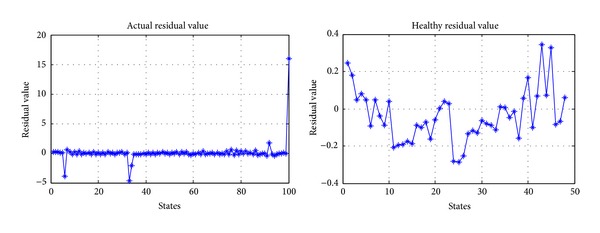
Actual residual value and healthy residual value of *R*_*e*_ and *R*_ct_ after dimensionality reduction.

According to actual residual value *R*_*X*_ and healthy residual value *R*_*L*_ of LIBs obtained by MSET, the anomaly detection for LIBs can be carried out based on SPRT. But according to the introduction of Section 4, firstly, the residual matrix should be preprocessed before anomaly detection based on SPRT. On the basis of (12), the residual matrix is transformed into one-dimensional vector by introducing the weight vector *X* = [0.5  0.5], shown in Figure 15, where the left figure is the actual residual value after dimensionality reduction and the right figure is the healthy residual value after dimensionality reduction.

So after that, the mean test of residual value will begin. Secondly, we formulate the following assumptions:

(19) $$\begin{array}{c} H_{0}\text{:}\;\text{mean}\;\mu_{0},\text{variance}\;\sigma_{0}^{2},\\ H_{1}\text{:}\;\text{mean}\;\mu_{1},\text{variance}\;\sigma_{2}^{0}. \end{array}$$

Thirdly, we select *α* = 0.001 and *β* = 0.005 and then get the upper limit of detection *B* = 995 and the lower limit of detection *A* = 0.005.

**Figure 16:**
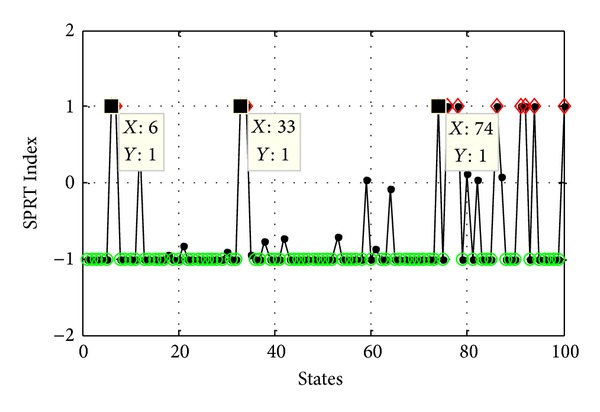
Result of AMM.

Lastly, in accordance with (14)–(18) and calculation steps of SPRT Index, the final SPRT Index of AMM is obtained, and the result of AMM is shown in Figure 16, where diamond represents the abnormal states (such as state 6, state 33, and state 74), dot represents the unable determined states and circle represents the healthy states.

**Figure 17:**
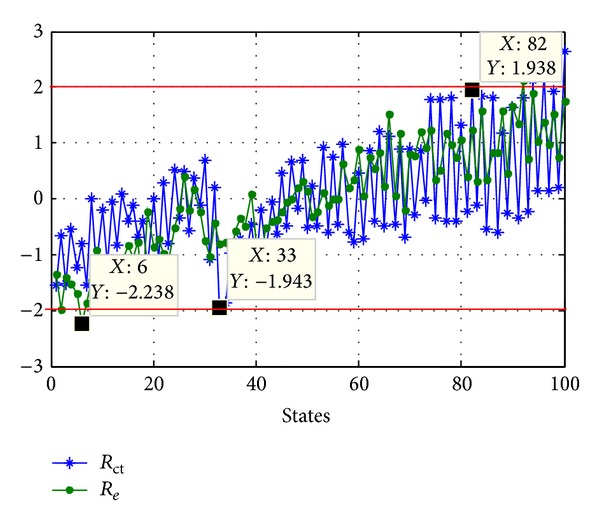
Result of TDM.

However, based on the data in Figure 11, the result of TDM is shown in Figure 17, where the abnormal states are state 6, state 33, state 82, and so on. Comparing these two results, we demonstrate that the AMM can detect not only the abnormal states outside the threshold (such as state 6 and state 33) but also the abnormal states in the threshold (such as state 74); furthermore, the AMM detects the abnormal state ahead of eight time states alarm than the TDM.

## 6. Conclusions

This research proposes a fault diagnosis method for key components of satellite, called AMM, based on the study of MSET and SPRT, which is developed by improving MSET and creatively combining MSET and SPRT. Due to the importance of satellite power system in the entire satellite system and the superiority of LIBs, we select the LIBs as the subject investigated. Then, after analysis failure of LIBs, we select *R*_*e*_ and *R*_ct_ as the key parameters of AMM. The AMM consists of state estimation based on MSET and anomaly detection based on SPRT. On the basis of fault diagnosis problems of LIBs in practical engineering applications, we improve the MSET and expound the key technologies of MSET at great length, that is, data normalization process, selection of training data *T*, selection and optimization of nonlinear operator, and structure of memory matrix *D*. And then according to the actual residual value and healthy residual value of LIBs that are got by state estimation, we have detailed illustration of the mean test of actual residual based on SPRT. Lastly, we conduct an example of AMM for LIBs, and, according to the result of AMM, we validate the feasibility and effectiveness of AMM by comparing it with the result of TDM.

The investigation in this paper is conducted from an engineering perspective. Extensive applications demonstrate that the model and algorithm are feasible, effective, and advanced. Studies on AMM can offer theoretical and technical guidance for fault diagnosis.

Recommendations for future work are threefold: (1) An in-depth analysis failure of LIBs should be conducted and more influencing parameters should be considered; (2) More studies on the key technologies of MSET and fault diagnosis methods should be carried out; (3) to serve as guidance for prognostics and health management using our methods.

## Figures and Tables

**Table 1:** Actual observation matrix *X*_obs_.

Time	*t*_1_	*t*_2_	*t*_3_	*t*_4_	*t*_5_	*t*_6_	*t*_7_	*t*_8_	*t*_9_	*t*_10_	…
*R*_ct_	0.078	0.088	0.078	0.085	0.078	0.084	0.078	0.084	0.078	0.083	…
*R*_*e*_	0.061	0.065	0.060	0.062	0.061	0.062	0.060	0.062	0.059	0.062	…
Capacity	2.035	2.025	2.013	2.013	2.000	2.014	2.013	1.968	1.968	1.957	…

**Table 2:** Observation matrix *X*_obs_ after data normalization process.

Time	*t*_1_	*t*_2_	*t*_3_	*t*_4_	*t*_5_	*t*_6_	*t*_7_	*t*_8_	*t*_9_	*t*_10_	…
*R*_ct_	−0.809	1.738	−0.939	0.897	−0.844	0.664	−0.927	0.655	−0.956	0.522	…
*R*_*e*_	−0.245	2.229	−0.586	0.540	−0.456	0.328	−0.905	0.300	−1.432	0.228	…
Capacity	1.291	0.909	0.466	0.464	−0.013	0.487	0.045	−1.202	−1.225	−1.635	…
